# The distribution of probability values in medical abstracts: an observational study

**DOI:** 10.1186/s13104-015-1691-x

**Published:** 2015-11-26

**Authors:** Bastiaan Ginsel, Abhinav Aggarwal, Wei Xuan, Ian Harris

**Affiliations:** Department of Orthopaedics, The Ingham Institute for Applied Medical Research, Liverpool, NSW Australia; Department of Biostatistics, The Ingham Institute for Applied Medical Research, Liverpool, NSW Australia

**Keywords:** p-hacking, Publication bias, p value

## Abstract

**Background:**

A relatively high incidence of p values immediately below 0.05 (such as 0.047 or 0.04) compared to p values immediately above 0.05 (such as 0.051 or 0.06) has been noticed anecdotally in published medical abstracts. If p values immediately below 0.05 are over-represented, such a distribution may reflect the true underlying distribution of p values or may be due to error (a false distribution). If due to error, a consistent over-representation of p values immediately below 0.05 would be a systematic error due either to publication bias or (overt or inadvertent) bias within studies.

**Methods:**

We searched the Medline 2012 database to identify abstracts containing a p value. Two thousand abstracts out of 80,649 abstracts were randomly selected. Two independent researchers extracted all p values. The p values were plotted and compared to a predicted curve. Chi square test was used to test assumptions and significance was set at 0.05.

**Results:**

2798 p value ranges and 3236 exact p values were reported. 4973 of these (82 %) were significant (<0.05). There was an over-representation of p values immediately below 0.05 (between 0.01 and 0.049) compared to those immediately above 0.05 (between 0.05 and 0.1) (p = 0.001).

**Conclusion:**

The distribution of p values in reported medical abstracts provides evidence for systematic error in the reporting of p values. This may be due to publication bias, methodological errors (underpowering, selective reporting and selective analyses) or fraud.

## Background

The relatively high incidence of p values immediately below 0.05 (such as 0.047 or 0.04) compared to p values immediately above 0.05 (such as 0.051 or 0.06) has been noted by the authors, and has been previously reported in samples of studies reporting effect sizes (Gøtzsche) and in psychology studies [1–4]. If p values immediately below 0.05 are consistently overrepresented, such a distribution may reflect either the true underlying distribution of p values or may be due to systematic error (bias) [1, 2]. This bias could be due to publication bias or (overt or inadvertent) bias within studies, as described below.

There is some evidence that, due to flexibility in the analysis and reporting of research data, the probability of finding a p value less than 0.05 can be increased by making use of “researcher degrees of freedom” [3]. Such degrees of freedom include (but are not limited to) variability in application of the inclusion and exclusion criteria, in the choice of independent and dependent variables in any analysis, and in the choice of analytical methods. Selective reporting of the research processes and findings makes such flexibility difficult to detect. The process of exploiting flexibilities in the analysis of research data has been labeled p-hacking [3, 5, 6]. This may represent a form of confirmation bias if researchers make use of the flexibility available to them to confirm their a priori beliefs.

It is also possible that differences in reported p values may represent publication bias, whereby studies with p values above 0.05 are less likely to be published (or submitted for consideration of publication) compared to studies with p values less than 0.05 [7–9]. The contribution to the over-representation of significant p values in the psychology literature, due to publication bias and the high proportion of underpowered studies, has been calculated previously [10]. If the reporting of p values in medical publications is biased in favor of values below 0.05, this would have consequences for clinical decision making, either from direct interpretation of the literature or via systematic reviews and guidelines [11].

We aim to estimate the distribution of all p values reported in medical scientific abstracts by an unbiased pictorial representation of the distribution.

## Methods

### Search strategy and selection criteria

With the goal of testing a large representative sample of the medical literature we used a random sample of all abstracts published in Medline in 2012 containing a p value. We chose 2012 as it was the most recent full year available at the time of the study. We used the following search string on 29 October 2013: “2012”.yr. and “p”.ab, limited to Abstracts and Humans. The search returned 80,648 abstracts and these were numbered consecutively. We generated 2000 random numbers between 0 and 80,649 with the random integer generator at http://www.random.org and selected the corresponding 2000 articles for inclusion in the study.

### Data extraction

We recorded the unique identifier, abstract text, and study type. Two investigators (BG and AA) independently extracted all p values from 500 abstracts, showing an inter-observer level of agreement of 94.2 %. For the remaining 1500 abstracts, data were abstracted by one (of two) investigators (750 each), and then checked by the other investigator. Inconsistencies between investigators were discussed with a third investigator (IAH).

### Statistical analysis

Many abstracts did not report a p value, but reported a range, usually “p < 0.05” or “p < 0.001”. Ranges of p values were excluded from the primary analysis. A separate study to withdraw these exact p values from their original full paper is currently being undertaken. All p values reported in the abstracts were counted within each interval with division of 0.01 and then plotted graphically. The underlying (expected) distribution of p values was estimated by an exponential curve. Chi square test was applied to compare the expected distribution versus the observed distribution. A significant p value (p < 0.05) derived from this Chi square test indicates an improbable observed distribution of the p values collected from abstracts. A separate analysis was performed for the study type randomised controlled trial (RCT) and also plotted graphically. The expected distribution in RCTs would be uniform if the null hypothesis is correct [12].

## Results

**Fig. 1:**
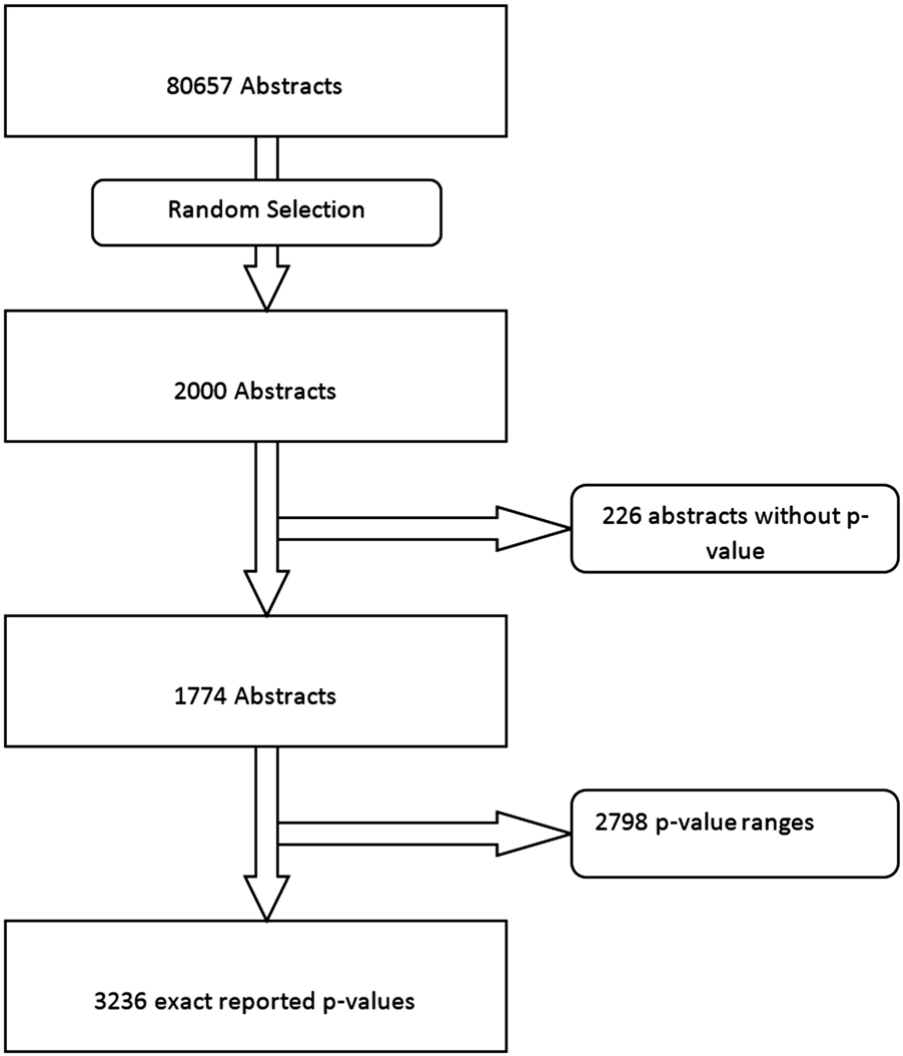
Flowchart of included abstracts

**Fig. 2:**
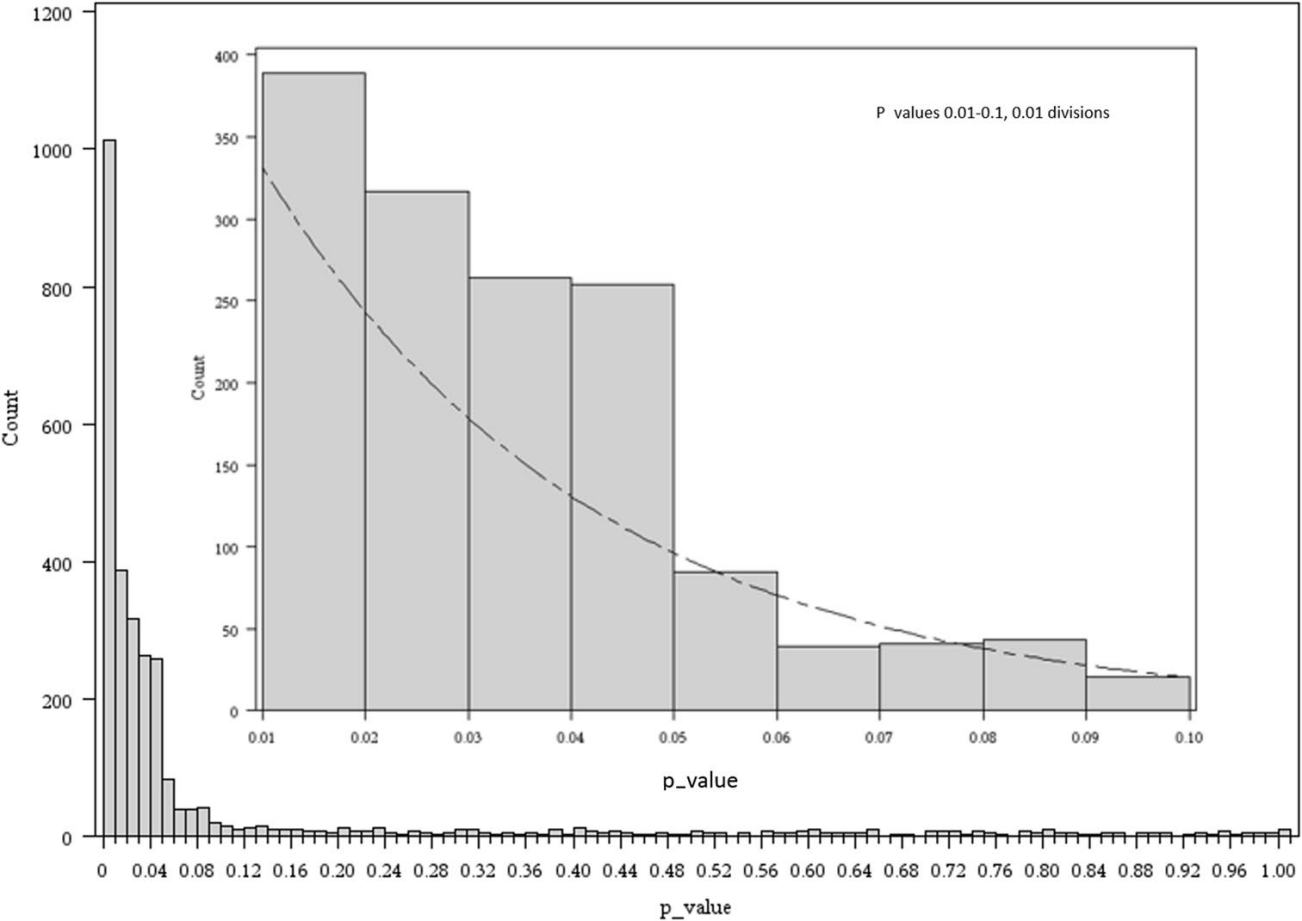
All p values between 0 and 1 are plotted in the *bottom* *graph*. The *inset* shows p values between 0.01 and 0.1 in 0.01 divisions

Two hundred and twenty-six abstracts out of the selected 2000, abstracts did not have a p value in their abstract and were excluded. Fourteen abstracts did not involve humans but were labeled as such by Medline. These were included, as they satisfied our a priori criteria of being labeled as human research in Medline and because this will allow more reliable replication of the study. The random selection and inclusion process is shown in Fig. 1. The 1774 included abstracts reported 2798 ranges (including 1069 p < 0.001 and 723 p < 0.05) and 3236 exact p values. The distribution of the 3236 exact p values is shown in Fig. 2. The inset in Fig. 2 is a magnification of the distribution of p values between 0.01 and 0.1. A relative over-representation of p values between 0.04 and 0.05 can be seen.

**Fig. 3:**
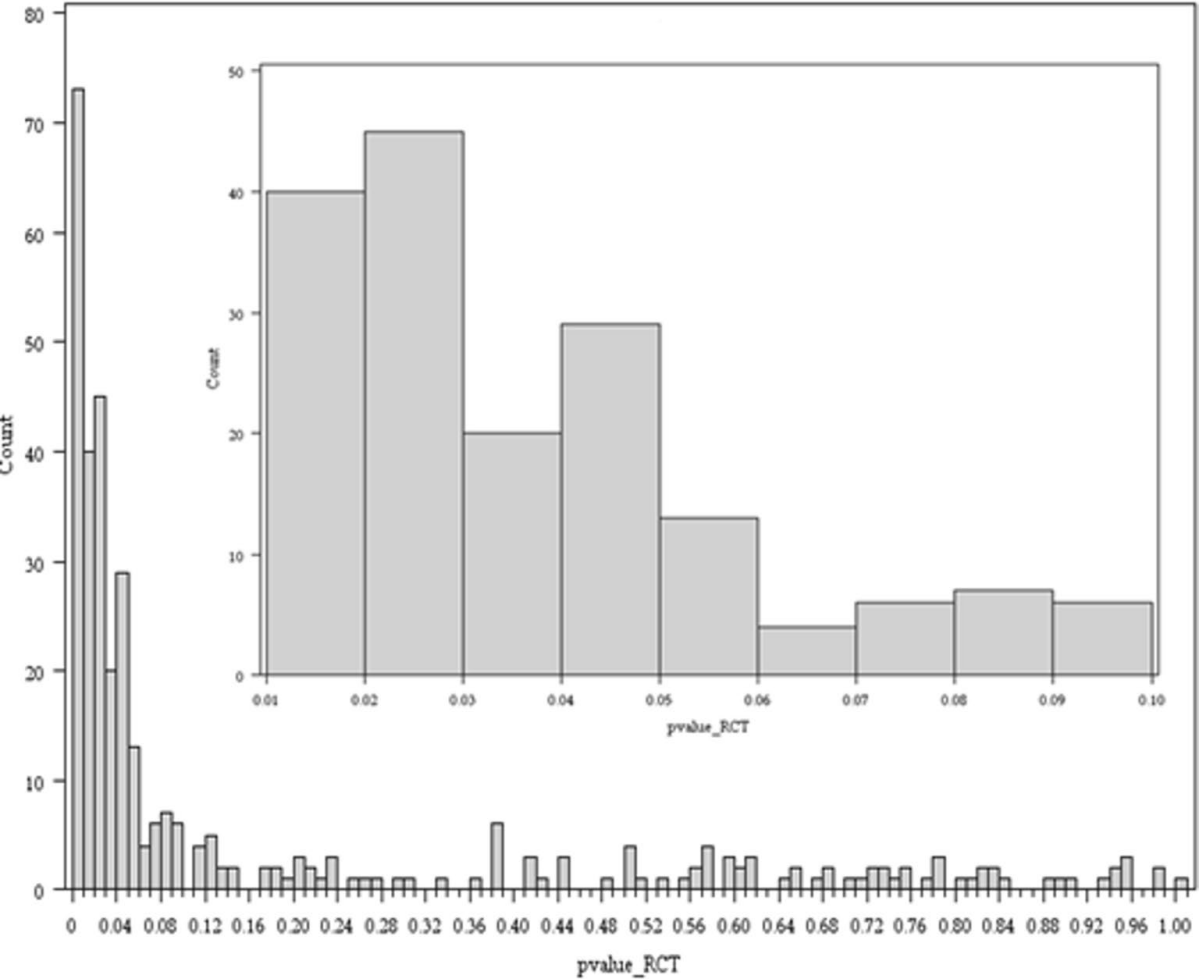
All p values from RCT type study between 0 and 1 are plotted in the *bottom* *graph*. The *inset* shows p values between 0.01 and 0.1 in 0.01 divisions

The probability of any p value in our study being less than 0.05 (including those given as ranges) was 82 % (4973/6034). There were 186 abstracts from RCTs that reported 350 true p values. The distribution of the exact 350 p values is shown in Fig. 3. The inset in Fig. 3 is a magnification of the distribution of p values between 0.01 and 0.1.

## Discussion

We demonstrated a biased distribution of p values in abstracts listed in Medline 2012, with an apparent increase in p values lying immediately below 0.05 relative to the frequency immediately above 0.05. This finding is likely to be evidence of p-hacking (biased analysis and reporting) or publication bias. We expected a more uniform distribution of p values for randomized trials, based on the assumption that these trials are done where equipoise exists, indicating that there is no expected difference between the two groups. However, the distribution of p values in this group was similar to the overall group.

Although the graphic presentation of the p value distribution in this study shows a clear picture, the weakness of this study is the lack of statistical analysis. There is no theoretical or calculated true distribution that can be used to test against. The decision to use all p values may have introduced some bias due to the inclusion of similar (related) p values from single studies. We also plotted the distribution of the first recorded p values, but found this to be similar to the distribution of all reported p values, and as the latter contained more data, we chose to report all p values. The p values used in this study were only the p values reported in the abstracts. This was chosen because many readers only read the abstract, the abstract is the only part of the article available in Medline, and because the most important p values are likely to be reported in the abstract [13, 14]. We excluded p value ranges, but this probably led to an underestimation of the bias around 0.05, as the reported range was commonly given as “p < 0.05” and including these was likely to have added to the number of p values immediately below 0.05. During data collecting we observed some errors in the Medline database. P values greater than one were reported and some papers labeled as human were in fact non-human. These errors were infrequent and were unlikely to have influenced the results. It is possible that, given that papers often contain many p values, significant p values in the manuscript are more likely to be reported in the abstract than values above 0.05. However, we consider this to be another form of selective reporting bias, as only the most important outcomes (such as any primary, patient-important outcomes) should be reported in the abstract, regardless of significance.

Gøtzsche commented on the distribution of p values between 0.04 and 0.06, noting a higher than expected number of values below 0.05 [2]. His study used first-reported p values in abstracts and noted a higher proportion of significant p values in non-randomised studies compared to randomised trials. Gøtzsche noted that the high proportion of significant findings in randomised trials is unexpected, given the need for equipoise (presumed equivalence of treatment options) in clinical randomised trials, which, if present, would lead to an unskewed (flat) distribution of p values. He also noted that many of the significant p values were incorrectly reported or analysed. The findings of Gøtzsche are consistent with the presence of bias in analysis and reporting and consistent with findings of our study. Masicampo et al., in a study of p value distribution in the abstracts of three major psychology journals, showed that there were more p values immediately below 0.05 than expected, based on the p value distribution in other ranges [1]. The distribution they found matches the distribution in this study. Jager and Leek looked at five major medical journals and their reported p values over a decade, however they only reported the distribution of p values less than 0.05 [15]. The distribution reported was similar to our distribution, although their study did not provide any information on the relative frequency immediately below and above 0.05. Simonsohn et al. suggested in 2013 to use a “p-curve”, a graphic p value distribution as a tool to evaluate if the literature on a certain topic has been influenced by publication bias or p-hacking. They declare that a right skewed p-curve is evidence of biased analysis or selective reporting [4]. Ioannidis also concluded that significant p values were over-represented in a review of meta-analyses of neuroleptic agents for schizophrenia [16]. Apart from publications bias and bias in analysis and outcomes reporting, Ioannidis added data fabrication as another possible cause of an over-representation of significant p values.

Distinguishing between publication bias and methodological biases (bias in analysis, selective reporting and data fabrication) is difficult. Funnel plot asymmetry, often interpreted as evidence of publication bias, can also be explained by these other forms of bias, as p values are artificially lowered and effect estimates exaggerated [17]. However, we consider bias immediately adjacent to 0.05 (as shown in our study) more likely to be due to methodological biases (working to push the p value below the level of significance), than due to publication bias (which applies to all p values below 0.05, not necessarily those immediately below 0.05). Methodological biases (rather than publication bias) leading to an over-representation of lower p values is also consistent with findings of effect estimate exaggeration in research [18–20] and with problems relating to the replication of significant findings in the medical literature [21–23]. Our study implies that the reporting of p values in human research is biased. Further research should explore predictors of bias in the distribution of p values, such as study type, methodology, study size, and journal type. In a later study, we aim to report on the distribution of p values described as ranges, such as “p < 0.05”, and to report on possible predictors of significance.

There is some evidence that the quality of reporting abstracts has improved over time due to initiatives such as CONSORT [24–26]. However, the reporting requirements only apply to some studies (such as randomized trials) and do not exclude the possibility of methodological biases and selective reporting. Unfortunately there are many more reasons why science has been incapable to self-correct [27].

Reports of statistical significance in medical research influence clinical decision making. Bias in such reporting should be considered when interpreting information from abstracts. Prevention of bias in reported p values would require open and complete reporting of research protocols and methods (to avoid analysis and reporting bias), adjusted analyses due to multiple testing (allowing for the increased probability of finding significance), and publication of all research (to avoid publication bias) [28].

## Conclusion

A biased distribution of p values in abstracts listed in Medline 2012 has been demonstrated, with an apparent increase in p values lying immediately below 0.05 relative to the frequency immediately above 0.05. This finding is likely to be evidence of p-hacking (biased analysis and reporting) or publication bias.
